# Improving implementation of tobacco dependence treatment practice in low and middle-income countries settings: a perspective from Jordan

**DOI:** 10.3389/frhs.2025.1696442

**Published:** 2025-12-17

**Authors:** N. Obeidat, A. Hatoqai, N. Mahmoud, S. Obeidat, S. Hammoudeh, F. Hawari

**Affiliations:** 1 Cancer Control Office, King Hussein Cancer Center, Amman, Jordan; 2 Pulmonary Medicine Section, King Hussein Cancer Center, Amman, Jordan; 3 Pharmacy Department, King Hussein Cancer Center, Amman, Jordan; 4 Ministry of Health, Amman, Jordan

**Keywords:** tobacco dependence treatment, smoking cessation, implementation science, low-to-middle income, Jordan

## Abstract

In Jordan, a Low- Middle-Income Country (LMIC) in the Eastern Mediterranean Region (EMR), tobacco use rates are among the highest globally. These alarming rates impose a huge economic and health burden and are exacerbated by cultural norms, societal misperceptions, and insufficient policy implementation. The tobacco epidemic is a multidimensional and complex one requiring multiple complementary solutions. One such solution is the availing of tobacco dependence treatment (TDT) services. However, establishing and maintaining TDT services can be challenging in resource-challenged countries. In this Policy and Practice Paper, we conducted a comprehensive critical analysis of Jordan's experience in initiating, expanding and maintaining TDT services, with the intention of providing insight which other LMICs seeking to establish TDT services can find useful. Our analysis is guided by the Consolidated Framework for Implementation Research (CFIR). Specifically, information was collected through both a desk review of the available evidence, and through expert insight from six healthcare practitioners directly involved in the establishment and/or implementation of TDT in Jordan. A CFIR assessment template was used to document the evidence and gather expert insights across the five CFIR domains (Innovation Domain, Outer Setting, Inner Setting, Individuals Domain, and Implementation Process Domain). Lessons learned and recommendations also were generated within each CFIR domain. Our findings, while presented in the context of Jordan as an LMIC, can be of use to other countries and settings with similar limited resources that will need to consider the adaptability and complexity of TDT, the broader policy and environmental setting within which TDT will be established, the physical and practice settings hosting TDT services, the potential stakeholders to engage in TDT establishment, and the changing implementation challenges faced when sustaining TDT services in an LMIC. Thus, our review can assist resource-limited countries planning or preparing to implement TDT services.

## 1 Introduction

### 1.1 Background and context

Tobacco use is a global public health problem which contributes significantly to morbidity and mortality. Across countries, disparities in tobacco use exist with regards to prevalence of tobacco and nonmedical nicotine use and rates of decline of use of these products; comprehensiveness of tobacco control regulations and their implementation; and access to services for tobacco and nonmedical nicotine cessation (1). The Eastern Mediterranean Region (EMR) in particular faces challenges: not only is the area unlikely to achieve the global target of a 30% reduction in tobacco use, but it is anticipated that some countries will face an increase in tobacco and nonmedical nicotine product use or else will see no change to the current dismally high rates of use of these products (1, 2).

The World Health Organization presents six policies that – if comprehensively implemented in a country – can curb the tobacco epidemic. These include Monitoring tobacco use and prevention policies; Protecting people from tobacco smoke; Offering help to quit tobacco use; Warning about the dangers of tobacco; Enforcing bans on tobacco advertising, promotion and sponsorship; and Raising taxes on tobacco (MPOWER policies) (3). Because nicotine dependence is a chronic, relapsing disorder, offering help to quit is essential in order to address the millions of tobacco and nonmedical nicotine users that cannot quit on their own (4). Tobacco dependence treatment (TDT) interventions are inarguably among the most beneficial and cost-effective public health interventions to avail in any country facing a tobacco epidemic (5). There is substantial evidence on the range of methods and interventions that can help smokers quit (5), and increasing research with regards to how to best implement such interventions (6, 7). However, most of the studies informing knowledge about TDT interventions and their implementation are generated by high-income countries. LMICs tend to suffer from various challenges that deter the establishment of TDT services, including limited resources and competing public health priorities, high rates of tobacco use even among healthcare practitioners, and limited availability of pharmacotherapies which tend to also be unaffordable even when available (8). Few studies shed light on the provision of TDT services in LMICs (9), and fewer yet provide details with regards to successful experiences in establishing TDT services.

Jordan, a LMIC that is located in the EMR, suffers from a very high prevalence of tobacco use. The latest Jordan Adults Tobacco Survey (JATS)^1^ in 2024 indicates that 71.2% of Jordanian males and 28.8% of females use at least one form of tobacco or nonmedical nicotine (cigarettes, waterpipe, heated tobacco, and electronic cigarettes). The high prevalence of tobacco use in the country has profound consequences. National statistics confirm that cardiovascular disease followed by cancers are the leading causes of death^2^. Studies have demonstrated the enormous opportunity cost of failing to control modifiable risk factors like tobacco use in Jordan in terms of additional years lived with disability and lower healthy life expectancy (10). The estimated economic impact of tobacco in the country annually was estimated to be (US$ 2.25 billion), 6% of Gross Domestic Product (2).

Despite disappointing observations regarding the prevalence of tobacco use in the country, Jordan has progressed in several tobacco control measures, including increasing tobacco taxation rates (to reach 78%); conducting large-scale media campaigns to educate the public; changes to improve – in theory – smoke-free legislations; bans on direct and indirect forms of tobacco advertising; and establishing TDT services which include both behavioral interventions and medications (short and long-acting nicotine replacement therapies and varenicline) (4). Jordan's expansion of its TDT services was first highlighted in the WHO's 2021 MPOWER report (11), and is a critical progression for a country where among males – 7–8 of every 10 males are smokers or vape, and where rates of smoking are showing a consistent increase in women.

A detailed understanding of how Jordan progressed in its TDT service establishment is valuable to share and can assist other LMICs as well as funding agencies seeking to enhance support for TDT services in LMICs. Jordan's continuing struggles with its tobacco epidemic while nevertheless being able to achieve some success in TDT represent a case study that others can find useful, particularly if presented in a structured framework using implementation science tools. In this paper we aim to provide insights with regards to TDT service establishment in Jordan and contribute to a broader literature on TDT implementation in low-resource settings by:

- Exploring the implementation of TDT services in Jordan using a structured implementation science tool.
- Highlighting the challenges and opportunities for improving TDT services.
- Providing recommendations for improving the implementation of TDT services.

### 1.2 Significance and potential impact

As an LMIC in the EMR, Jordan's experience and challenges with establishing and maintaining TDT services in low-resource settings offers a unique and under-represented [in the scientific literature] perspective that can contribute to global efforts to understand how to approach TDT solutions in low-resource settings with unique cultural and social contexts. Moreover, presenting Jordan's experience using an implementation science framework can encourage other LMICs to approach the planning and evaluation of TDT services in a similarly structured manner.

## 2 Methods

### 2.1 Defining TDT interventions

TDT interventions include various behavioral and pharmacological treatments offered in different formats and intensities, and through potentially several health or behavioral professionals. Behavioral treatments can be offered through individual, group, or telephone counseling, and through digital applications; the kinds of behavioral therapies provided can also include cognitive behavioral therapy (CBT), motivational interviewing (MI), acceptance and commitment therapy, and incentive-based interventions; and there are seven globally recognized pharmacotherapies [short and long-acting nicotine replacement therapies (NRTs), varenicline, bupropion and in many countries now cytisine] that are also used to augment behavioral treatments (5, 12).

In Jordan, TDT interventions are provided by physicians who are largely certified tobacco treatment specialists, and involve face-to-face individual counseling using behavioral counseling (MI with some elements of CBT), as well as pharmacotherapies, including varenicline, bupropion, and NRTs.

### 2.2 Framework selection

Implementation science frameworks are valuable tools to improve our understanding of how interventions can be established, implemented, evaluated and sustained. Such frameworks provide a mechanism to present the complex, multi-level factors that influence how an intervention works (13), and can enable different entities and countries to align and harmonize how they share their experiences in implementing critical interventions such as TDT services. We employed the Consolidated Framework for Implementation Research (CFIR) to guide document retrieval and analyses, and to obtain critical expert insight with regards to how TDT services evolved and continue to operate in Jordan (14). The CFIR has been widely used to document and evaluate TDT services in various settings (15, 16), and is composed of five main domains which are defined in the context of this assessment as follows:

- **Innovation domain:** evidence-based Tobacco Dependence Treatment (TDT) interventions being implemented in Jordan that are in line with international best practices.
- **Outer setting:** the broad socio-political, economic, cultural, and policy environment context that affects the successful adoption of TDT services at an institutional level.
- **Inner setting:** the characteristics of institutions that are responsible for the implementation of TDT in Jordan.
- **Individuals' domain:** the roles and characteristics of those who are directly involved or affected by TDT implementation.
- **Implementation process domain:** the activities and strategies used to implement TDT services.

The domains are further composed of additional constructs that collectively cover the multi-level factors that shape the implementation of the innovation (14), and are discussed in detail in subsequent sections.

In addition to the CFIR, the Expert Recommendations for Implementing Change (ERIC) project's framework was used to generate actionable recommendations. The ERIC taxonomy includes 73 defined implementation strategies that were synthesized from the literature and can potentially be used to implement interventions (categorized into nine themes: using evaluative and iterative strategies, providing interactive assistance, adapting and tailoring to context, developing stakeholder interrelationships, training and educating stakeholders, supporting clinicians, engaging consumers, utilizing financial strategies, and changing infrastructure) (17).

### 2.3 Approach

#### 2.3.1 Working group establishment

Experts included a Health Services Researcher, a Public Health Specialist, a Pharmacist, and three clinicians who also work as Tobacco Treatment Specialists and have overseen the establishment of TDT services (both governmental and non-governmental) and/or currently work within them. The team also included TDT-related evaluation and research expertise. Collectively, the team represented the two core entities in Jordan providing TDT services.

#### 2.3.2 Desk review

The desk review involved two experts who conducted two independent reviews of both published literature as well as governmental documents and media releases related to Jordan and smoking cessation or tobacco dependence treatment (Pubmed and Google search engines were used). The aim of the desk review was to identify key information, and priority was given to national studies and data. Governmental websites, where a number of reports are housed, were also searched to identify documents or national reports (largely in Arabic) produced in Jordan and related to health data. WHO Framework Convention on Tobacco Control (FCTC) MPOWER reports also provided important information with regards to the status of tobacco control and tobacco dependence treatment in the country.

#### 2.3.3 Consensus meetings guided by the CFIR

Iterative consensus meetings were conducted with six experts (18). A CFIR-structured template was prepared and employed to organize information related to TDT in Jordan. The experts independently noted the status of each construct in Jordan as it pertained to TDT. Over an eight-week period, regular meetings and document exchanges and reviews were conducted to compile the feedback from each expert per CFIR domain. Specifically, per domain, key constructs were analyzed and experts participated in a series of structured discussions to compare assessments and discuss their views. During meetings, any further verbal feedback not included by experts was documented by two experts on the team. These group discussions resulted in a collective understanding and documentation of the evolution and implementation of TDT in Jordan. After iterative discussions of drafts, a final document was reviewed and refined. Finally, specific recommendations were formulated to strengthen future implementation efforts, and offer lessons that could benefit other countries seeking to enhance TDT using the taxonomy proposed by the ERIC project (17).

## 3 Critical examination of TDT implementation in Jordan

### 3.1 Innovation domain

#### 3.1.1 Innovation evidence-base and innovation source

Relatively few studies related to the efficacy of TDT interventions have emerged from Low and Middle-Income Countries (LMICs) (19–22), but the evidence-base regarding the general effectiveness of Tobacco Dependence Treatment (TDT) is well-established in the global literature (5, 23–27). Provision of TDT interventions is therefore recommended by leading organizations and experts (3, 12, 28).^3^ The widespread recognition of TDT as a critical service by such entities, as well as the availability of a set of core competencies for TDT provision^4^ have accelerated efforts of LMICs such as Jordan to establish TDT services.

In addition to global perspectives that have driven the uptake of TDT, the presence of credible, local expertise in Jordan has been essential in shaping the development of TDT skills and services. The King Hussein Cancer Center (KHCC), the only specialized comprehensive cancer treatment center in Jordan, has long been a leading stakeholder in national and regional efforts to promote TDT (29). In 2008, the Center established a TDT clinic that would provide intensive counseling and pharmacotherapy to cancer patients. The clinic's establishment was an ethical obligation to encourage cancer patients to quit in order to reap the full benefits of their therapy and also served as an opportunity to build capacity in TDT. What began as a modest service gradually expanded over the years, both in staffing and in the number of clinics. Simultaneously, the efforts of the clinic became recognized both regionally and globally and enabled the establishment and implementation of TDT training programs. Eventually, KHCC was able to achieve accreditation by the Council for Tobacco Treatment Training Programs (CTTTP) in 2017^5^^,6^, gaining further credibility as an innovation source. In addition, as the service grew, data were generated to document the experiences and outcomes of KHCC in treating tobacco dependence, lending further credibility to both KHCC as a local innovation source, and TDT as a service (22, 30–32). Thus, when the Jordanian Ministry of Health (MoH) was able, in subsequent years, to embark on a dramatic expansion in TDT clinics in Primary Healthcare Centers^7^, KHCC's insight and training activities were critical in ensuring the expansion was accompanied by a growth in qualified practitioners to provide TDT. Similarly, other smaller college clinics and Municipal clinics in Jordan have turned to KHCC to become trained in TDT service establishment. KHCC represents the potential, in LMICs, for non-governmental entities trusted by the community to adopt the promotion of evidence-based TDT and supplement governmental efforts.

#### 3.1.2 Innovation relative advantage

For an intervention to demonstrate relative advantage, it must do so relative to other available interventions or current practices (14). In Jordan, prior to establishing any TDT interventions, no solutions were available for supporting smokers seeking to quit. This facilitated the case for availing TDT services, despite the well-recognized challenges of achieving high long-term abstinence rates [even with intensive treatment in controlled environments, long-term abstinence rates may not exceed 30% (24)]. However, within the available interventions for TDT, the relative advantage of different medication regimens or behavioral approaches is not definitive, and Jordanian TDT guidelines do not recommend any specific regimen over another. Given the already limited options available for TDT, LMICs seeking to establish TDT services should encourage the use of all available TDT medications and evidence-based behavioral approaches to the greatest extent possible.

#### 3.1.3 Innovation adaptability, complexity and trialability

The adaptability of TDT in Jordan, particularly as a result of resource constraints and cultural differences, is a characteristic that is important to take into account. Intensive TDT interventions can and should be adapted to different cultural, economic, and health system contexts (33, 34). Interventions can be delivered by a wide range of healthcare providers including, but not limited to, physicians, pharmacists, nurses, and nutritionists (12). TDT interventions also can be offered at various levels of intensity, with more intensive treatment exposure generally yielding better outcomes (35). For example, the benefits of interventions ranging from monotherapies over a few weeks to intensive combination therapies over extended periods, and a dose-response for counseling have been demonstrated (12, 35). Interventions are also guided by patient-reported withdrawal symptoms and thus must also be adaptable at the patient-level.

In Jordan, TDT interventions have been adapted in more than one context, including:

- TDT services are provided in Arabic, and the presence of an accredited TDT training program that communicates to Arabic practitioners while using local examples and contexts has further facilitated the application of the TDT evidence-base in a practical manner in clinics. Counseling involves culturally and linguistically adapted Motivational Interviewing techniques with some aspects of Cognitive Therapy (36, 37), rather than conventional Cognitive Behavioral Therapy.
- TDT providers in Jordan also have had to adapt treatment during periods of medication shortages by taking advantage of fewer options for medications or in some situations using behavioral therapy alone. Medications, even when available, are also limited to specific pharmaceutical forms and dosages (for example, despite being licensed, nicotine lozenge is currently not sold in Jordan). In smaller clinics (colleges and Municipal clinics), medications, if available at all, are limited to nicotine gums.
- Adaptability of TDT also takes place at the level of the target group of smokers. At KHCC, TDT counseling is culturally attuned and contextualized to the cancer journey and experiences. In smaller college clinics, TDT service providers tailor counseling to young adults and their college experience.

TDT adaptability in the context of Jordan is both advantageous and challenging. It is advantageous in that there is no one-size-fits-all solution, and resource-appropriate evidence-based measures exist and can vary. Conversely, it is challenging because decision makers and payers expect specific and predefined estimates of the required resources for TDT establishment and sustainability. It is important to reiterate to decision makers that TDT interventions are variable, and even when not fully implemented in a manner similar to that observed in high-income settings, can still be feasible, acceptable, and cost-effective.

The subject of TDT's adaptability is related to its complexity (38). TDT interventions can potentially include multiple components that add to intervention complexity (39). Adaptability in terms of varying components and methods of implementing each component can in turn make the standardization, implementation and integration [within existing systems] of TDT more complex.

In LMICs like Jordan with severely strained primary healthcare systems, and in specialized centers with competing services and other health priorities, integrating and connecting yet another service such as TDT without prior assessments of how the service will be implemented within the system can have negative unintended consequences, regardless of how seemingly easy it may be to adapt interventions at the patient level. Thus, piloting the implementation of TDT interventions is essential. Well-designed pilot studies or implementation trials conducted with the clear purpose of reporting on key implementation measures have not been conducted in Jordan. At KHCC, TDT services grew gradually, and to date need to be continually revisited in light of changes in the local healthcare system. At the MoH, services were expanded and announced without prior piloting.

#### 3.1.4 Innovation design

At the MoH, primary care practitioners can refer smokers to the TDT clinic, and otherwise healthy smokers can directly request an appointment. Conversely, TDT at KHCC is bundled within the cancer care package and is promoted as a core supportive service. The concept of TDT is relatively new in Jordan, but both KHCC and the MoH promote their TDT services to the lay public and to practitioners who can refer to the clinics, and TDT-focused content is highlighted in each entity's website and tobacco-related materials^8^^,9^^,10^^,11^^,12^. Nevertheless, a widescale and more structured approach to promoting a tangible set of services would benefit TDT implementation in the country.

With regards to tools that have been developed to support TDT services, most currently available TDT materials in Jordan are in Arabic and include provider quick references (e.g., brief advice steps), patient brochures and booklets (covering tips to quit, visuals on the health effects of smoking, and a timeline of the benefits of quitting), and clinic posters. These vary in clarity and design and are largely adapted from international resources but use locally familiar terms when translated into Arabic. There is certainly room for improvement, particularly with regards to availing more dynamic demonstrations of TDT and more testimonials that can be used to educate both the lay community and healthcare practitioners. While testimonials from ex-smokers have the potential to motivate smokers to quit and have been developed by some entities (e.g., at KHCC), they are still not widely used. The use of digital tools for TDT in Jordan also is limited. Few entities have piloted mobile applications for TDT^13^^,14^, but these are still not fully operational and are not integrated into healthcare systems.

It is also important to highlight that the establishment and promotion of an accredited TDT training program [through KHCC] has played a pivotal role in presenting TDT as a recognized concept, a professional skill, and an essential service.

#### 3.1.5 Innovation cost

Despite TDT being one of the most cost-effective interventions in public health (40, 41) and despite studies demonstrating cost-effectiveness within the Jordanian context (42), translating these studies to approved budgets to support TDT interventions in Jordan has not been straightforward. As documented in the general literature, cost-effectiveness represents only one dimension of an intervention, and it is not unusual for cost-effective interventions to be dismissed or only partially supported as a result of budgetary concerns (43). In Jordan, healthcare spending is an ongoing concern^15^. Pharmaceutical spending alone was estimated at 1.12 billion USD in 2022 (30% of healthcare spending)^16^. Based on public unit prices for medications in Jordan, an approximate cost of a 12-week combination therapy for TDT ranges from approximately 500 to 700 USD. Given that smokers pay more for cigarettes annually^17^, one would think that smokers trying to quit would be willing to bear the cost of TDT services. In Jordan, this has rarely been the case and smokers are generally unwilling to pay for TDT services. Thus far, most TDT services in the country do not impose substantial out-of-pocket costs on beneficiaries.

### 3.2 Outer setting

**Figure 1:**
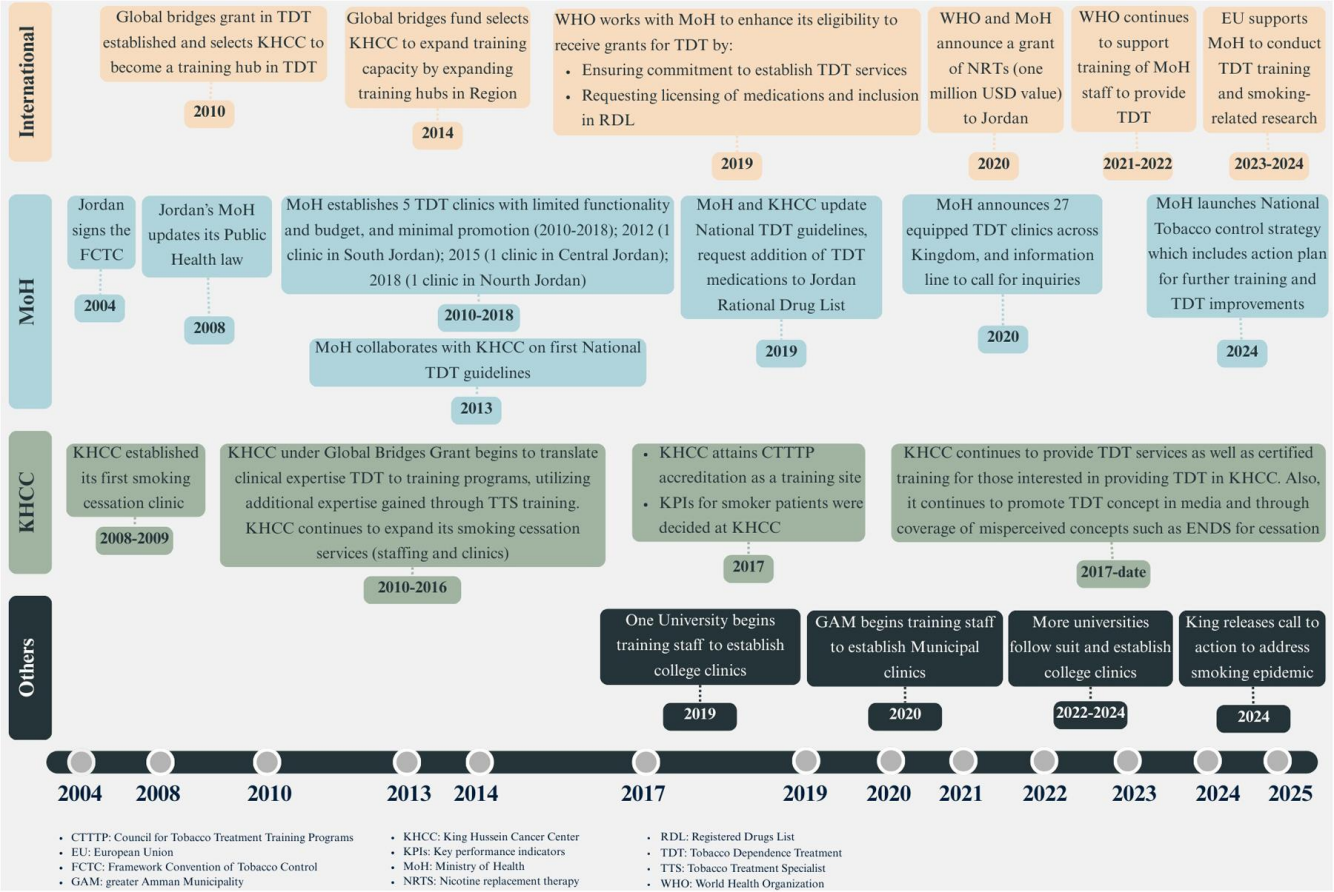
Key events and stakeholders that have influenced the tobacco dependence treatment outer setting in Jordan.

As depicted in Figure 1, the Outer Setting that has shaped the availability and growth of evidence-based TDT services in Jordan has included various international and national drivers as well as stakeholders. In addition, the overall socioeconomic conditions faced by Jordanians, and community norms and expectations with regards to tobacco use and TDT, have further shaped the demand for TDT.

#### 3.2.1 Policies and laws

Jordan was one of the first countries to ratify the World Health Organization (WHO) Framework Convention on Tobacco Control (FCTC) in 2004 (4). Jordan's subsequently revised tobacco control laws and policies also are strongly aligned with the principles of the FCTC and the MPOWER policy framework. Biannual MPOWER reports tracking the global progress across MPOWER policies reveal that over time, Jordan has implemented the six MPOWER measures, albeit to varying degrees (4). Conversely, Jordan is one of the few countries that is demonstrating a worrying trend in tobacco and electronic cigarettes (vaping) use (44), with rates steadily increasing over the past decade^18^^,19^ (45).

Jordan's tobacco epidemic prompted a call to action by the country's King Abdullah II^20^, which in turn facilitated the official launch in 2024 of a long-awaited National Tobacco Control Strategy. Although the strategy had undergone several rounds of development and revision in previous years, its official launch in 2024 marked the point when a tobacco control action plan was formally disseminated to all government entities for collaborative implementation. The Strategy is framed around the FCTC and MPOWER policies of the WHO and was developed by the MoH in collaboration with various stakeholders who advocated for the inclusion in the Strategy of several measures related to the provision and expansion of evidence-based TDT services in the country.^21^

With regards to the Offer policy of MPOWER [TDT programs], Jordan ranks in the highest level of implementation (“National quit line, and both NRT and some cessation services fully or partially cost covered”) (4). A hotline is available which provides information regarding the locations and times of public TDT clinics but does not offer any form of counseling. With regards to medication-related policies, nicotine patches, lozenges, gums, varenicline and more recently bupropion are registered medications in Jordan's Food and Drug Administration (JFDA), while nicotine nasal spray and oral inhaler are available at KHCC under a special import license. The MoH is currently in the process of facilitating the acquisition of nicotine nasal spray. It also is relevant to note that while bupropion is available for use by TDT physicians at KHCC, it is limited in use to psychiatrists at the MOH and not to TDT practitioners in MoH TDT clinics.

National guidelines for TDT, first issued in 2013 and updated in 2019^22^, are also available and emphasize the importance of providing, at a minimum, brief advice. The guidelines also provide an overview of the core elements of TDT. These guidelines have been a helpful starting reference but now require updating to address in a more in-depth manner intensive tobacco and electronic cigarettes (vaping) cessation interventions and newer evidence with regards to the use of TDT medications.

#### 3.2.2 Financing

There has been more than one form of TDT financing in Jordan's history:

- Prior to 2021, there was no clear financing scheme available to cover TDT services in the few undersupplied TDT clinics in the MoH. Financing was erratic, and when available was limited. Medications could not be purchased on a large scale through the government's centralized medication procurement system. This form of financing was unsustainable and hindered the establishment of a credible and reliable TDT service.
- In 2020, a large grant covering NRTs facilitated the scaling up of TDT services, and 27 TDT-providing MoH clinics (free of charge) were announced throughout the country.^23^ Although the grant was not a sustainable financing mechanism, it ensured the scaling up of TDT services and the availability of medications to a substantially larger number of beneficiaries for a period of time. The grant also prompted the integration of TDT medications into the government's centralized medication procurement system and commitment from the MoH to sustain TDT services.
- Subsequent to the grant, TDT service provision is currently ongoing in Jordan's public clinics but is dependent on the availability of adequate governmental financing and the often-protracted process of governmental medication procurement. Thus, it has not been unusual to observe intermittent medication supplies in the country (this problem is not unique to TDT medications).
- At KHCC, and since TDT service establishment in 2008, the costs of TDT have been included within cancer treatment coverage and ultimately financed by the government of Jordan (other smaller cancer treatment centers in the country do not offer cessation services as part of cancer care). Smokers from the community and who are not patients can use the service but must bear the costs of treatment.
- Other less common forms of limited financing also exist. In one university, a separately funded initiative (under the Alliance of Jordanian Universities Against Tobacco and Smoking^24^) to support multiple tobacco control activities, covers the cost of training tobacco treatment specialists and securing a limited amount of nicotine gums annually. A similar setup of local funds exists in the Municipal clinic in Amman.
- Occasionally, private corporations have partnered with TDT service providers to cover TDT services to groups of employees as a form of incentive. While not a consistent mechanism of financing, it may be useful to other LMICs to take note of.

To date, transparent conversations about the long-term financing of national TDT services in Jordan have not been had. Hypothetically, treating 200,000 smokers (a humble figure given smoking rates in the country) could cost at least 100 million USD (roughly 9% of pharmaceutical spending). While these figures are broad estimates, they underscore the importance of approaching any proposal for establishing or expanding TDT services with caution. It would be unrealistic to expect that such a portion of pharmaceutical spending could be dedicated exclusively to TDT medications. In addition to the cost of medications, the cost to establish TDT services includes staff salaries, providing training to staff, producing materials and tools, purchasing equipment (e.g., CO monitors and mouthpieces), and the cost of additional infrastructure needed. Currently, TDT costs are covered through the government (*see Financing in Outer Setting Domain*) for smokers seeking governmental cessation services, and to cancer patients and KHCC employees utilizing KHCC's TDT clinics. Private insurance companies do not cover any form of TDT, despite the potential role that they can play in improving access to TDT (5).

The issue of affordability and financing of TDT is critical, particularly with regards to the implications of inconsistently available resources and supplies on patient retention, quality care, and service credibility. Difficult but essential discussions are needed to (a) ensure feasible financing solutions are available, (b) prioritize TDT provision within subgroups of smokers, and (c) include more specific resource-appropriate recommendations in local TDT guidelines.

#### 3.2.3 Local conditions; local attitudes

Despite having a relatively comprehensive Public Health Law, data have consistently indicated that in Jordan, enforcement of tobacco control laws is weak and limits the extent to which an environment and culture conducive to TDT can be achieved. Tobacco use is highly prevalent and normalized, and using electronic cigarettes (vaping) is becoming a concerning trend (46). Jordan's public health law generally prohibits smoking in public places and bans the marketing and promotion of tobacco and electronic cigarettes (vaping products)^25^. However, the majority of Jordanians report substantially high (exceeding 65%) rates of exposure to secondhand smoke in public places^26^^,27^. Cigarette prices remain relatively affordable despite high taxation rates^28^, and tobacco products are accessible to children^29^. Waterpipe smoking is a seemingly inseparable part of social life (47), and is particularly prevalent in young adults (48–50). Moreover, there is a high prevalence of poly tobacco use (48). It is therefore not surprising that approximately 39% and 84% of smokers in Jordan begin smoking before the age of 17 and 24, respectively^30^. In such a culture and environment, smokers attempting to quit face a demotivating environment that heightens their risk of relapse. Concerningly, quit attempts appear to be declining. In 2014, 2019, and 2024, 62.8%, 44.2% and 37% (respectively) of smokers in Jordan had [unsuccessfully] tried to quit (45).^31^^,^^32^

Nevertheless, there are other aspects of the Jordanian culture that can be leveraged to counter these concerning trends. In 2014, 2019 and 2024, roughly 20%, 28% and 73% (respectively) of smokers in Jordan were advised to quit smoking by their physicians (45),^33^^,34^ indicating a growing recognition among healthcare providers of the importance of addressing tobacco use in clinical practice. In addition, earlier studies suggest that religion, a key component of Jordanian culture, can be a viable tool to encourage cessation (51). The Ministry of Awqaf, Islamic Affairs and Holy Places in Jordan (a government body responsible for managing Islamic religious affairs) officially prohibits using electronic cigarettes (vaping) and smoking in all its forms^35^^,36^, and a substantial proportion of Jordanians also are aware of the religious stance against smoking^37^. The extent to which this can be capitalized on needs further exploration.

Finally, with regards to other positive cultural changes, over the recent years, several efforts are being made by tobacco control advocates and health organizations to promote the concept of tobacco control and TDT as a component of tobacco control. The MoH, KHCC and various Non-Governmental Organizations (NGOs) such as Tobacco Free Jordan (La Lil Tadkheen), the Alliance of Jordanian Universities Against Tobacco and Smoking (AJUATS), the Jordanian Anti-smoking Society, Royal Health Awareness Society (RHAS), and Eastern Mediterranean Public Health Network frequently promote the concept of TDT at public health events and health days and in professional meetings related to preventive health TDT services. At Petra University, the university which pioneered the establishment of a college TDT clinic, a precedent was set that prompted other universities within the AJUATS to follow suit.

#### 3.2.4 Partnerships and connections

Partnerships and connections have the potential to spearhead the establishment of TDT programs. LMICs vary in the governmental, private, academic and international collaborations that exist and how they can be leveraged. In Jordan, since ratifying the WHO FCTC in 2004, many partnerships and connections were established with national, regional and international entities to support its TDT efforts (Figure 1). The key health authority, the MoH, collaborates with the WHO to receive technical guidance for implementing the FCTC and MPOWER package, including “O” for offering help to quit tobacco. Non-governmental clinical entities (for example KHCC) have partnered in the past with global entities^38^ in order to establish certified TDT training programs. Agreements such as healthcare quality-related ones [e.g. the Joint Commission International (JCI)'s disease-specific accreditations for cancer centers] also have been leveraged to avail screening and referral services to cancer patients who smoke.

National partnerships in Jordan have generally included collaborations for establishing and sustaining TDT services, for availing TDT medications, for generating tools and guidelines for TDT, for building capacities in TDT, and for raising awareness on and generating demand for TDT. For example, the MoH has a long-standing collaboration with clinical entities like KHCC, where the latter provides support through capacity building, consultancy in designing tools, and monitoring and evaluation. Jordan's healthcare accrediting body (HCAC) collaborates with the MoH and other non-governmental entities to integrate Smoking Cessation Brief Advice into accreditation standards, and to promote smoke-free entities.

Partnerships have also been critical in developing technical documents necessary to support TDT services. The MoH in Jordan partnered with KHCC's TDT program to provide the required technical evidence to the Jordan Food and Drug Administration (JFDA) in order to expand the list of licensed TDT medications and advocate for their inclusion in the National Rational Drug List. This particular process was also facilitated by the WHO's expansion of its Essential Medicines List to include more TDT medications^39^ (52). Non-governmental entities also collaborate with one another to amplify smoking cessation related messages and knowledge. Such collaborations have emphasized the relevance of TDT across a wide variety of local contexts, further validating the importance of smoking cessation as a public health matter.

Partnerships in Jordan have occasionally also included the private sector. For example, more than one private corporation has in the past partnered with TDT service providers to cover TDT services to groups of employees. The cumulative value of these partnerships cannot be understated. Both international and national relations have collectively led to the recognition of TDT in Jordan as an essential service that is worth allocating resources towards, and worth continually improving and sustaining.

#### 3.2.5 External pressure and critical incidents

Over the past two decades, multiple incidents have affected the implementation of TDT interventions in Jordan. Wars and prolonged conflicts in the Middle East have resulted in an increased influx of refugees and increased the demand for subsidized healthcare services (creating challenges in maintaining consistent delivery of preventive services), and have changed national priorities in different sectors^40^. In addition, changes in leadership at the MoH have affected tobacco control in the country and at times have resulted in a shift in public health and TDT-related priorities on a national level.

The COVID-19 pandemic was another significant event that influenced the implementation of TDT in Jordan. The impact varied across implementing institutions: while the MoH expanded and prioritized TDT services during the pandemic, KHCC encountered substantial internal and pandemic-related challenges. These challenges led to the disruption of TDT services at KHCC and a subsequent reduction in the clinics' operational capacity.

In 2021, a global shortage of varenicline occurred as a result of voluntary recalls of the drug (53). Given the limited number of medication options available for TDT, this rapidly impacted TDT services. At the time, there were few approved generic versions of varenicline in most markets (and none in Jordan). The absence of varenicline as a therapeutic option severely impacted the overall provision of TDT worldwide (54), and certainly in Jordan. Relatedly, the subsequent production of generic varenicline in Jordan was a critical progression that helped facilitate the gradual resumption of varenicline use.

Particularly in the context of an oncology center, the announcement of the scope of the National Cancer Institute's Moonshot program was important (55). While the program had no direct impact on TDT delivery, the inclusion of TDT in cancer care as one of the subjects among a list of critical oncology matters highlighted the urgency of TDT in cancer settings. Those at KHCC involved in TDT were able to leverage the Moonshot program to underscore that the adoption and continuing operation of TDT services was not only critical for patients but was also a means of enhancing KHCC's international standing as a cancer center committed to delivering comprehensive, person-centered care.

### 3.3 Inner setting

This include primarily MoH health clinics and KHCC as a tertiary [cancer] center. Smaller scale TDT services include clinics in college campuses and municipal health clinics (namely Greater Amman Municipality ''GAM'' serving the capital of Jordan). Characteristics of the inner setting include structural, relational, communications-related, cultural, situational, and resource as well as knowledge-related. In Jordan, there is great variability in the capacities of healthcare institutions to deliver TDT, with differences in infrastructure, manpower, communications, and institutional culture.

#### 3.3.1 Structural characteristics

In theory, the structural requirements to establish TDT services within an entity include the physical space, the technological systems, and the necessary staff (14):

- With regards to space, the distribution of physical space varies significantly throughout institutions. Both the MoH and KHCC's TDT clinics are scheduled during designated days and times when existing clinic spaces are available and not in use for other medical services. At the MoH, the size and level of comfort of available privacy and space for TDT varies across MoH Centers. The few colleges that have established clinics have designated a space for use only for TDT or lifestyle modification counseling. The former approach (no permanent space for TDT) has facilitated TDT establishment because no unique space requirements were required but, conversely, has also limited the available days and times for TDT services which compete with other medical services. The latter approach (establishing a space only for TDT) has ensured that the TDT clinic could be operational more frequently, but committing to a new space is not practical in many entities.
- With regards to staffing and operations, the now operational 29 TDT clinics at the MoH run approximately two to four clinics a week on separate days (three to five hours per clinic)^41^. These clinics are typically run *after* other primary care clinics have been completed or on days where no primary care clinics are running. With regards to the number of MoH clinics, although the 29 cover the 12 governorates of the country, they are likely insufficient given the current status of tobacco use. A hypothetical and rough approximation demonstrates why: in Jordan, there are roughly 7.7 million residents aged 15 or older, of whom 3.3 million smoke and 1.2 million have tried to quit.^42^ The 29 clinics collectively provide 323 h of service weekly (approximately 15,200 h a year). If a smoker roughly needed a total of 135 min of physician time across the cessation treatment pathway [within a range proposed in the literature (28)], this would equate to over 2 million hours of needed physician time a year. While by no means reflective of actual numbers, the calculation is intended to provide perspective on the adequacy of MoH clinics that are available in the country. The currently operational clinics at the MoH have been a critical and positive progression in Jordan and should be regarded as an opportunity to understand and finetune TDT services for subsequent expansion or else more strategic prioritization of these services.
  Entities that operate a smaller scale than the MoH also vary in their frequency of operation. At KHCC, TDT clinics typically operate 3–4 h a day, two to four days a week. Frequency of clinics is influenced by physician availability to manage the TDT clinic but given the much smaller number of smokers (who are cancer patients) being treated at the clinic, TDT services at KHCC face less demand in comparison to MoH clinics.
- In addition to clinic space, clinic location has also impacted TDT services. TDT clinics at the MoH are accessible if smokers are willing to visit these typically congested clinics and wait. At KHCC, a physically large entity, the proximity of the TDT intake service to other essential services was an advantage. After the relocation of these essential services to a more distant location within the Center, there was a notable reduction in number of patients visiting the TDT clinics. This observation highlights the importance of visibility of TDT services and, in entities where it is applicable such as healthcare centers and hospitals, the potential value of integrating TDT within other clinical services rather than as a standalone service that other services refer to. In colleges, the availability of a clinic on campus has been instrumental in giving students a more practical option (than visiting busier MoH clinics) for TDT.
- With regards to technological systems, nearly all health institutions in Jordan utilize electronic health records (EHR). However, the breadth and consistency of documentation vary widely (56). For example, in MoH clinics, the use of a combination of paper-based and electronic documentation is still common. At KHCC, electronic smoking-related data are collected through Center-wide screening forms that must be completed for patients, through TDT clinic-specific notes when patients receive TDT, through unstructured physician notes when smoking is mentioned outside the TDT clinic, and through a clinic database that was designed to document TDT-related measures in an in-depth manner. Decision-support tools such as automated alerts and TDT stepwise protocols are absent, but mechanisms are currently being sought to change this.

It is relevant to also note that across TDT clinics in Jordan, there are no virtual or telehealth-based TDT services available. While this may limit accessibility for individuals who cannot visit facilities in person, a brief pilot experience at KHCC during the COVID-19 pandemic left providers with an unfavorable impression of the potential for remote counseling/patient follow-up. The experience suggested that remote services may not, for now, be desired by communities. On the other hand, KHCC also successfully developed a pharmacy call center service to provide counseling on medication use. The contrasting experience suggests that telehealth may be useful within specific components of TDT management, and requires piloting to better understand.

#### 3.3.2 Resource and knowledge-related characteristics

TDT medications (primarily NRTs and varenicline) are available in most MoH centers and at KHCC. Medication procurement at the level of the MoH is through a centralized process, but there are challenges with maintaining a consistent supply of these medications, particularly in MoH clinics where demand is higher given the patient load in governmental clinics. Within KHCC, medications can be purchased through a process independent of governmental medication procurement, and this has facilitated the more consistent availability of medications at the Center.

With regards to the TDT workforce, there are some differences across institutions. Across Jordan, physicians dedicated solely to TDT are rarely observed. At the MoH, TDT services are led by family physicians who conduct the screening, counseling, and documentation of patient information (on occasion, they are also asked to conduct training on brief advice to other physicians to ensure that referral to the MoH's TDT clinics takes place). Pharmacists are responsible for dispensing TDT medications, but have not taken a recognized role yet, nor do they typically receive TDT-related training. Largely physicians are trained to be tobacco treatment specialists and run TDT clinics, and they often report being overloaded with competing work (other clinic services). Physicians who cover other services and also provide TDT certainly can enrich the patient's counseling by virtue of their insight into other medical conditions, but competing priorities often mean less time and support is dedicated to the TDT clinic. Non-physician healthcare practitioners at the MoH (e.g., pharmacists and nurses) could assist physicians in many aspects of TDT but do not.

These challenges also exist in non-governmental TDT clinics. However, at KHCC, although TDT physicians cover other services, the TDT clinic also employs nursing staff. Both physicians and nurses are trained in TDT, and the latter provide indispensable support in the TDT clinic by screening and orienting patients; conducting patient initial, CO measurement, and follow-up assessments; and coordinating all functions in the TDT clinic. In addition, the Center is currently expanding the responsibilities of both pharmacists and Clinical Nurse Coordinators (CNCs) across the hospital to include a role in counseling on cessation.

A valuable opportunity to both build and disseminate knowledge with regards to TDT in Jordan has involved knowledge brokers. Neal et al. summarize the characteristics of knowledge brokers as those who “build relationships…to help facilitate the flow of information, and to provide the necessary human element of interaction, communication, mentoring, skills building and knowledge sharing required for effective evidence-based health promotion practice… they lead to the development of ideas, or management of a particular common interest shared” (57). Both governmental and non-governmental TDT clinics in Jordan work closely with the Cancer Control Office (CCO) at KHCC, the CCO acting as a knowledge broker. The CCO supports and collaborates with TDT clinic staff in research, training and resource development, and in promoting the TDT service across diverse stakeholders. The CCO also communicates with TDT staff with regards to critical TDT-related policy developments beyond the Center.

Similarly, at the MoH, the Health Awareness and Communication Directorate advocates for policies and administrative decisions that facilitate the work of TDT physicians in MoH clinics. The Directorate also promotes the concept of TDT in mass media campaigns and ensures TDT-related measures are reflected in any action plans that can be linked to preventive health.

#### 3.3.3 Relational connections and communication

Coordination within each institution with regards to TDT varies. The MoH faces a challenging situation, given the number of staff at the MoH and across 12 governorates of the country, and given the absence of clear communication pathways to ensure TDT-related information is disseminated effectively.

At KHCC, despite the ease of within-Center communications and a clear mechanism to coordinate referrals between TDT clinic staff and physicians, knowledge gaps still exist that are related to the TDT service, its value, and how it can be accessed (58, 59). The TDT service has been promoted in a brief training module available online to all healthcare practitioners, but more needs to be done. An institution-specific guideline is currently being developed which will delineate the roles and responsibilities of core departments and staff. The guidelines will be disseminated in a manner that ensures broad reach across the institution. Proactive and continuous formal communications (emails from higher administration; grand rounds) are also sought when needed.

#### 3.3.4 Culture

The extent to which a TDT service aligns with an institution's mission and culture is important in shaping the delivery of TDT services. At KHCC, a person-centered approach to care has been adopted, making it easier to promote TDT as a service of value for both employees and patients who smoke. KHCC staff can benefit from free TDT services, and these (as well as the center's smoke-free policies) are included in all new employee orientations. KHCC also promotes clinical research as a key institutional output, and the TDT service – in collaboration with others in the institution – includes research in its activities. This aspect has been helpful in further highlighting the value of the TDT service. The TDT service also enabled KHCC to attain accreditation of its TDT training, and training in general is also a key element of KHCC's mission. In other institutions, the culture might not be as supportive of TDT services and activities if the latter are not clearly tied to other institutional priorities. This is particularly the case when the institution prioritizes disease management over preventive services.

### 3.4 Individuals domain

We define the “Individuals domain” in the context of this assessment as the roles and characteristics of those who are directly involved or affected by TDT implementation. This includes individuals who provide TDT, those who facilitate its implementation, and those who receive it. Understanding the wide spectrum of roles and unique characteristics of individuals has been important in tailoring TDT interventions and TDT promotion in Jordan.

#### 3.4.1 Leaders

The early development of TDT practices in Jordan was driven by a number of leaders at the MoH (Minister and Heads of Directorates such as the Health Awareness and Communication Directorate) and KHCC (Chairperson of the board of trustees of the entire Center and Foundation, the Center's Director General, and the Head of the TDT clinic); and subsequently in smaller sites such as colleges (led by the Alliance of Jordanian Universities Against Tobacco and Smoking and University Presidents in colleges that established clinics) and Municipal clinics.

At the MoH, initial efforts were insufficient as a result of both the limited number of clinics and shortage of medications. At KHCC, with a smaller clinic and few staff, but available medications and high-level leadership support, efforts extended beyond service delivery and included data generation, expertise enrichment, training program development, promotion of cessation concepts in the community, and advocacy to ensure the service grew in staff and clinic number. Subsequently, when the MoH was able to secure resources to expand the number of TDT clinics, both MoH and KHCC leaderships supported a collaborative approach through training, and addressing existing regulatory challenges related to finally including TDT medications in Jordan's Rational Drug List (to facilitate national procurement).

Moreover, these leaders invested in the creation and development of qualified teams committed to tobacco control in general and TDT in specific. Several individuals involved in TDT were able to receive competitive training scholarships in tobacco control and advanced TDT training. These opportunities were valuable not only for strengthening expertise but also for highlighting the significance of local TDT experiences in gaining regional and international recognition, and for encouraging further leadership support.

#### 3.4.2 Implementation leaders and facilitators

A broad range of individuals in Jordan are involved in the promotion or implementation of TDT, albeit with different levels of influence. Academia (universities and university hospitals) have played a role in promoting the concept of TDT to students, training staff in TDT, inviting staff involved in TDT to share their local experiences with students and staff, and generally recognizing that TDT is an important health topic to be supported. NGOs have been instrumental in promoting the concept of cessation, with some subsequently playing a direct role in establishing a few TDT clinics in college campuses. At KHCC, the CCO has supported the TDT service since its inception, providing technical support to design and deliver TDT training programs, monitor and analyze TDT data, advocate for TDT inclusion in related health projects, and promote TDT in the community.

A critical component in facilitating TDT delivery is the existence of a network that recognizes the value and presence of the service and refers smokers to it. The MoH has not evaluated referral patterns to the clinic, but at KHCC, evaluations have revealed that knowledge gaps and poor referral practices have existed (58, 59). The integration of referrals within hospital accreditation standards has helped address some of these concerns, but continual efforts remain needed to ensure the service is accessible to other healthcare practitioners and the latter are indeed utilizing it.

In addition, other staff are pivotal to TDT implementation. At KHCC, pharmacists play a key role in facilitating the implementation of TDT services. Close coordination between TDT staff and pharmacy management is essential to ensure the timely procurement of adequate quantities and appropriate forms of TDT medications. Furthermore, with the expanding role of clinical nurse coordinators (CNCs) as gatekeepers of information and appointments for patients, there has been a focus on training CNCs in the importance of brief advice and referral to the TDT clinic.

#### 3.4.3 Innovation deliverers

The motivation of practitioners to provide TDT has differed significantly across entities. In larger entities such as the MoH and based on observations on engagement during KHCC's TDT training programs, this has varied by clinic within the MoH; and has been influenced by level of training, age, clinical load, and interest in the subject of TDT. At KHCC, only motivated physicians who have expressed interest and are able to allocate part of their clinical load to TDT have been involved in providing TDT services. Finally, at college campuses, only those interested in TDT have been nominated to receive training and establish clinics. Direct incentives are not provided to TDT service providers although recognition can be highly beneficial in boosting the motivation to pursue a career in TDT. With regards to expertise, most providers involved in TDT are certified through KHCC's accredited training program. This has helped standardize the information and skills being shared with practitioners.

#### 3.4.4 Innovation recipients

Studies have indicated that although the proportion of smokers who have tried to quit has gone down^43^^,44^, the number of smokers in the country who would potentially use TDT services is substantial. Contextualizing TDT benefits is bound to be useful in reaching smokers in their unique situations, as has been the case in KHCC. Patients are educated about the benefits of cessation relevant to their specific cancer as documented in the literature (60). Demonstrating that local results also confirm these benefits has also assisted TDT staff in framing messages about cessation (22). Nevertheless, high cessation rates are challenging to achieve in cancer patients being seen at KHCC; long-term abstinence rates reach approximately 22% (22). Evidence generated indicates that patients at KHCC require more support, more knowledge-sharing, and more interventions that can build self-efficacy to quit; and are subject to challenging triggers that can jeopardize their cessation attempts (61).

Currently, there are no public data available to understand the perspectives of beneficiaries of the MoH's TDT services.

### 3.5 Implementation process domain

When TDT services were implemented, various steps were taken that are outlined below.

#### 3.5.1 Teaming

Teaming both among entities and within them was essential to establish TDT services. Among entities, guidelines were developed, and training arrangements were established to facilitate standardized knowledge and practices to the greatest extent possible. Within KHCC, leadership, implementers, and innovation deliverers have worked jointly to ensure that TDT services continue to operate smoothly. It is important to note that teaming is dynamic and, when opportunity allows, additional groups with potential to add value to TDT services are always being sought.

#### 3.5.2 Assessing need and context

Given the absence of any TDT interventions, pre-implementation assessments of need and context were not performed. Nevertheless, over the years, studies have been conducted in different entities to assess the perceptions of both patients and providers and have consistently confirmed a need for TDT services as well as a need for structured training to meet that need (58, 59, 62, 63). Systematic and recurring assessments to evaluate changing perceptions that can impact implementation of TDT are not frequently conducted, a weakness that requires addressing in Jordan's TDT settings.

#### 3.5.3 Planning

Entities involved in TDT had delineated specific roles and responsibilities for team members, and a database to track patients was established at KHCC. The database required the identification of various patient-related TDT measures, including sociodemographic characteristics, smoking patterns, withdrawal symptoms, and cessation experiences and outcomes (measures tracked at the MoH were not as detailed but included some aspect of each of these constructs). Anticipating these measures before establishing TDT services helped guide planning around how TDT services would be implemented and the goals they intended to achieve.

#### 3.5.4 Tailoring implementation strategies

Various implementation strategies have been used to implement and sustain TDT services, many of which have been cited in the literature (6, 7, 64, 65). Supplementary Table S1 describes how several implementation strategies were tailored to meet Jordan's needs and enhance TDT operations.

## 4 Actionable recommendations

Drawing on Jordan's experience with TDT service establishment, the following practical recommendations are suggested that apply both to Jordan and other LMICs seeking to establish or expand TDT.

### 4.1 Innovation domain

Identify all potential stakeholders that can potentially be involved in TDT service establishment, promotion, evaluation, and research and knowledge generation. Accordingly, convene a team of credible experts that can

- Produce a master document that showcases the value of TDT in the context of the LMIC and that highlights the potential cost-effectiveness of TDT relative to more familiar interventions in the community. The document can be used to showcase the importance of TDT to practitioners and decision-makers.
- Utilize national data and statistics to estimate approximate figures regarding potential TDT demand and the scope and reach of TDT services that would be needed.
- Plan feasibility and acceptability studies in the community to ensure that proposed TDT innovations (such as quitlines) are worth investing time and effort to advocate for.

### 4.2 Outer setting

- Review and document the key international organizations and agreements (e.g., FCTC) that promote TDT and use these to further highlight the importance of TDT services in international policies relevant to the country or setting.
- Frame TDT as a core aspect of tobacco control policies and ensure that those involved in TDT have sound knowledge in local tobacco control laws and policies and can advocate for their enforcement (e.g., regulations related to smoke-free laws, advertising bans, packaging requirements, and age restrictions, to reduce the accessibility and social acceptability of tobacco and nicotine products).
- Engage opinion leaders (including religious leaders), NGOs, and local groups to support advocacy efforts, dispel myths about tobacco use and cessation, and strengthen social support networks for quitting tobacco.
- Use national epidemiological data and global evidence-based guidelines to make the case for prioritizing tailored TDT interventions to high-risk populations (e.g., pregnant women, adolescents, dual/poly tobacco users, waterpipe smokers), while preserving core components for effectiveness.
- Establish a mechanism to regularly review/update with stakeholders (preferably including authorities responsible for medication registration and practitioner credentialing) available TDT guidelines to ensure that the guidelines follow the best practices in TDT but are also implementable in the country. Guidelines should also include core indicators that should be measured when any TDT service in the country is established.
- Address financial constraints and the potential for irregular medication supplies by securing sustained funding and streamlining supply chains for all first-line pharmacotherapies. Although medications are not the only element of intensive TDT, their use is crucial, and their absence has implications not only with regards to the patient's potential to succeed, but also with regards to patient retention, service credibility, and medication waste due to initiation of therapy and subsequent [and often abrupt] discontinuation of care.
- Innovate in how TDT is promoted in awareness campaigns regardless of campaign size, so that messages regarding cessation are culturally tailored but also novel in their approach to avoid message fatigue.
- Conduct proactive outreach and advocacy about the value of TDT at professional events and educational forums to continuously reinforce its importance in specific clinical contexts.
- Advocate for the institutionalization of TDT interventions by embedding them into routine health packages in entities where this is possible (e.g., insurance companies).

### 4.3 Inner setting

- Conduct structured assessments of TDT services barriers and facilitators (including patient, provider, and organizational levels), exploring cost, stigma, co-morbidities, and social norms to ensure that TDT services remain effective, relevant, and meet the needs of the target population. Such assessments should cover entity and context-specific factors but also include core elements to enable comparisons across entities in order to determine the viability of TDT service establishment and customization. The TDT literature is well-developed and contains numerous measurement tools [for example (66, 67)]. These should be taken advantage of.
- Establish a mechanism to regularly review/update with stakeholders in the entity implementing TDT available local TDT guidelines to ensure that the guidelines align with national guidelines (if available), follow the best practices in TDT, and take into account practical issues related to TDT implementation within the entity.
- Use epidemiological data available within the entity to make the case for prioritizing tailored TDT interventions to high-risk beneficiaries receiving care in the entity (e.g., comorbid patients, specific age groups in the entity).
- Design TDT intervention protocols that are visually easy to follow and delineate roles and responsibilities clearly, so that providers referring to TDT services and those within TDT services can integrate them within their daily practices.
- Invest in training and building capacities of non-physician health professionals (pharmacists, nurses, allied health workers) to support the delivery of intensive and brief TDT interventions, which also can increase accessibility and reach to a wider range of smokers. In a country with high smoking prevalence, even among healthcare practitioners, it is difficult to find nonsmokers who are motivated to provide TDT. Broadening the pool of practitioners engaged in TDT is therefore essential.
- Explore the potential for private clinics and community pharmacies to serve as extended points of TDT service delivery through collaboration on offering brief advice, TDT medication dispensing and counseling, and referral to comprehensive TDT services. This can be particularly beneficial in underserved areas.
- Develop culturally and linguistically tested and suitable materials within the entity and increase promotional efforts to raise smokers' awareness and demand for TDT services by using multiple media platforms. Relatedly, ensure that TDT service providers are aware of all media messages related to TDT in anticipation of demand surge.
- Ensure that proposals to expand TDT services are based on both qualitative and quantitative assessments of current services' scalability and evidence of successful implementation.
- Advocate for leadership engagement within healthcare organizations to allocate resources, staff time, and training to improve and sustain TDT delivery, by highlighting the value of TDT in the context of specific department outcomes (e.g., surgical outcomes, cardiovascular outcomes). Relatedly, per department or service, identify disease or service-specific guidelines and organizations that have included TDT within their scope (e.g., National Comprehensive Cancer Network, American Diabetes Association Standards of Care).
- Explore the nature of medical records in the entity and how they are typically used and propose feasible ways to integrate TDT measures (prompts for referral as well as measures related to TDT service delivery and patient outcomes) within the medical record.
- Support TDT within the organizational culture through financial (if available) and non-monetary incentives such as recognition, public acknowledgment, honorary titles, and opportunities for specialized training.

### 4.4 Individuals domain

- Provide standardized capacity-building opportunities for healthcare providers (HCPs) to improve their knowledge, skills, and confidence in delivering evidence-based TDT, and include refresher courses.
- Address the smoking prevalence issue among HCPs themselves by availing TDT to them. For example, at KHCC, employees are able to access TDT treatments free of charge to ensure that
- Increase HCPs' awareness of available TDT services and promote referral practices, especially in primary care settings.
- Raise awareness among patients about the importance of TDT and available treatment options through both clinical interactions and community engagement and testimonials when possible.
- Consider cost-sharing of TDT services if services are currently free of charge to ensure patient commitment and generate a mechanism to finance even if in part some of TDT.

### 4.5 Implementation process domain

- Establish a multidisciplinary team in the institution (including Nursing, Pharmacy, Internal Medicine, Statistical Units managing data) whose members can act as institutional champions and support the TDT service's promotion and implementation; and who can facilitate TDT-related communications across departments.
- Develop and implement adaptive, data-driven TDT implementation plans with clear goals, timelines, monitoring, and feedback mechanisms to guide continuous quality improvement.
- Employ participatory methodologies involving patients, providers, community leaders, and policymakers to tailor TDT services and tools used to promote TDT services, in order to enhance TDT relevance, acceptability and uptake.
- Pilot TDT interventions as well as follow-up forms using iterative Plan-Do-Study-Act (PDSA) cycles to optimize delivery and identify best practices before larger-scale implementation.
- Invest in a streamlined data-collection platform or service to collect and continually track TDT-related measures (both aggregate and patient-specific). Given the rapid progression in digital tools, such services have become more affordable and accessible.

## 5 Discussion

Jordan's experience in establishing TDT services offers important learning experiences for other LMICs and low-resource settings. The provided recommendations align with broader observations made in the literature with regards to enhancing TDT services in LMICS (8, 9, 68).

It is important to note that Jordan continues to face certain challenges in sustaining and expanding its TDT services. Efforts across the domains of the CFIR outlined in the paper need to be continually tracked and, to the greatest extent possible, enhanced. Nevertheless, key take home messages with regards to Jordan's experience that can provide insight to other LMICs are reiterated below.

International progress in TDT and tobacco control has encouraged (and pressured) many countries to consider TDT interventions more seriously. External organizations also serve as a motivator to showcase that even small-scale TDT interventions, when well-resourced and monitored, can prove valuable. Conversations about TDT across the global community should be encouraged as much as possible. Jordan's experience has demonstrated that such conversations have played a key role in shaping the development of TDT. LMICs should leverage existing international relations to the greatest extent possible.

The concept of TDT, while it continues to grow in Jordan, remains one that needs to be actively promoted as an innovation, despite having value that is comparable to, if not greater than, other less debated interventions in the community such as the use of statins and antihypertensives in heart disease (69–71). Other LMICs seeking to establish TDT services should be prepared to continually expend effort to highlight TDT and its importance, and communicate evidence-based numbers strategically using current evidence-based knowledge.

It is also imperative that TDT experts be well-versed in general tobacco control issues in the country. For example, in Jordan, the tobacco industry continues to unabashedly promote its products through different channels and mechanisms in order to perpetuate nicotine addiction and hinder cessation efforts (46). Those involved in TDT must be aware of the abundance of products and misperceptions related to these products and accordingly promote evidence-based TDT.

Despite the various difficulties an LMIC country may face, the establishment of TDT services is achievable even in challenging contexts, such as in Jordan, a country with one of the highest global rates of male smoking, and where TDT runs counter to prevailing cultural norms. Customized solutions can be crafted provided that the tobacco control community in general and the TDT community in specific, are always aware of the greater role they play across all domains of TDT implementation.

## Footnotes

1 Ministry of Health, Jordan Adult Tobacco Survey 2025. Factsheet. Available upon request.

2 Ministry of Health. Jordan Cancer Registry Cancer Incidence in Jordan – 2022. Available from https://moh.gov.jo/EN/List/Newsletters

3 European Network for Smoking and Tobacco Prevention. Tobacco Dependence Treatment Guidelines 2025. Available from https://ensp.network/wp-content/uploads/2025/05/2025-Guidelines-For-Treating-Tobacco-Nicotine-Dependance-English-v2.pdf

4 Association for Treatment of Tobacco Use and Dependence (ATTUD) Core Competencies for Evidence-based Treatment of Tobacco Dependence. Available from https://www.attud.org/assets/docs/Standards.pdf

5
https://www.ctttp.org/

6 King Hussein Cancer Center Tobacco Dependence Treatment Training Program. https://www.khcc.jo/en/tobacco-dependence-treatment-training-program

7 Jordanian Ministry of Health (MoH). Tobacco Dependence Treatment Clinics MoH Website MoH [updated June 11, 2025]. Details of the MoH TDT clinics in Jordan and their contact information.]. Available from: https://www.moh.gov.jo/AR/Pages/smoking.

8 King Hussein Cancer Center Smoking Cessation Clinic. https://www.khcc.jo/en/tobacco-dependence-treatment-clinic

9 Ministry of Health. Educational leaflets and briefs on smoking. Available from: https://www.moh.gov.jo/AR/Pages/%D8%A7%D9%84%D8%AA%D8%AF%D8%AE%D9%8A%D9%86 and https://www.moh.gov.jo/AR/ListDetails/%d9%85%d8%b9%d9%84%d9%88%d9%85%d8%a7%d8%aa_%d8%b9%d8%a7%d9%85%d8%a9_%d9%88%d8%b5%d8%ad%d9%8a%d8%a9/1045/20

10 King Hussein Cancer Center. Cancer Awareness and Outreach Activities. Educational materials available from https://www.khcc.jo/ar/awareness

11 Jordanian Ministry of Health Communication and Awareness Directorate Facebook page. https://www.facebook.com/HCADJO/?locale=ar_AR.

12 King Hussein Cancer Center. Cancer Awareness and Outreach Activities. Educational materials available from https://www.khcc.jo/ar/awareness

13 Naffasy app. https://www.instagram.com/naffasyofficial/

14 Jordan News Agency, June 3, 2025. JFBPW Launches Free Platform to Help Smokers Quit. Available from https://petra.gov.jo/Include/InnerPage.jsp?ID=72172&lang=en&name=en_news.

15 Jordan Times, May 15, 2025. Health spending in Jordan reaches JD2.67b, exceeding int'l, regional averages. Available from https://jordantimes.com/news/local/health-spending-jordan-reaches-jd267b-exceeding-intl-regional-averages

16 Jordan National Health Accounts 2020-2022. Available from https://moh.gov.jo/EN/List/Newsletters

17 UNDP, WHO. Jordan Tobacco Control Investment Case. Available from https://www.undp.org/jordan/publications/jordan-tobacco-control-investment-case

18 Jordan National Stepwise Survey (STEPs) for Noncommunicable Diseases Risk Factors, Jordan 2019. https://www.moh.gov.jo/Echobusv3.0/SystemAssets/5f91caaf-c257-4806-837c-b1028147b692.pdf

19 Ministry of Health, Jordan Adult Tobacco Survey 2025. Factsheet. Available upon request.

20 King Abdullah II Official website. Media Center, Aug 28, 2023. King says countering dangerous impact of smoking a priority, urges protecting school students. Available from https://kingabdullah.jo/en/news/king-says-countering-dangerous-impact-smoking-priority-urges-protecting-school-students

21 Jordan National Strategy to Control Tobacco and Smoking in all its forms, 2024 - 2030. Available upon request in Arabic from https://mol.gov.jo/.

22 Jordan National Tobacco Dependence Treatment Guidelines, 2019. Available from https://www.khcc.jo/uploads/2019/10/khccbookletsmokingaddiction.pdf

23 Jordan Times, July 11, 2020. WHO supports Health Ministry with about $1m to help thousands of smokers quit smoking. Available from https://jordantimes.com/news/local/who-supports-health-ministry-about-1m-help-thousands-smokers-quit-smoking

24 Alliance of Jordanian Universities Against Tobacco and Smoking Celebrates Its 10th Anniversary and Showcases Achievements. https://uop.edu.jo/en/news/pages/25-61.aspx

25 Tobacco Control Laws. Legislation by Country/Jurisdiction - Jordan. Available from https://www.tobaccocontrollaws.org/legislation/jordan/summary

26 Jordan National Stepwise Survey (STEPs) for Noncommunicable Diseases Risk Factors, Jordan 2019. https://www.moh.gov.jo/Echobusv3.0/SystemAssets/5f91caaf-c257-4806-837c-b1028147b692.pdf

27 Ministry of Health, Jordan Adult Tobacco Survey 2025. Factsheet. Available upon request; 2025.

28 Tobacconomics Cigarette Tax Scorecard: Spotlight on Jordan (2022). Available from https://www.economicsforhealth.org/files/research/804/jordan-scorecard-second-edition-policy-brief-final.pdf

29 Tobacco Free Jo & Cancer Control Office, King Hussein Cancer Center. Protecting Jordanian School Children from Tobacco Advertising Tactics Report (2023). Available from https://haqqi.s3.eu-north-1.amazonaws.com/2023-08/Protecting%20Jordanian%20School%20Children%20from%20Tobacco%20Advertising%20Tactics%20Report%202023.pdf

30 Ministry of Health, Jordan Adult Tobacco Survey 2025. Factsheet. Available upon request.

31 Ministry of Health, Jordan Adult Tobacco Survey 2025. Factsheet. Available upon request.

32 Jordan National Stepwise Survey (STEPs) for Noncommunicable Diseases Risk Factors, Jordan 2019. https://www.moh.gov.jo/Echobusv3.0/SystemAssets/5f91caaf-c257-4806-837c-b1028147b692.pdf

33 Jordan National Stepwise Survey (STEPs) for Noncommunicable Diseases Risk Factors, Jordan 2019. https://www.moh.gov.jo/Echobusv3.0/SystemAssets/5f91caaf-c257-4806-837c-b1028147b692.pdf

34 Ministry of Health, Jordan Adult Tobacco Survey 2025. Factsheet. Available upon request.

35 Jordanian Iftaa' Board. Determination of status of smoking in Islam (determination 1299). Available from https://www.aliftaa.jo/brief-fatwas/1299/-%D9%85%D8%A7-%D8%AD%D9%83%D9%85-%D8%A7%D9%84%D8%AA%D8%AF%D8%AE%D9%8A%D9%86

36 Jordanian Iftaa' Board. Determination of status of electronic cigarettes and waterpipe in Islam (determination 3436). Available from https://aliftaa.jo/research-fatwas/3436/%D8%AD%D9%83%D9%85-%D8%A7%D9%84%D8%A3%D8%B1%D8%AC%D9%8A%D9%84%D8%A9-%D9%88%D8%A7%D9%84%D8%B3%D9%8A%D8%AC%D8%A7%D8%B1%D8%A9-%D8%A7%D9%84%D8%A5%D9%84%D9%83%D8%AA%D8%B1%D9%88%D9%86%D9%8A%D8%A9

37 Ministry of Health, Jordan Adult Tobacco Survey 2025. Factsheet. Available upon request.

38
https://globalbridges.org/

39 Application for the inclusion of cytisine on the who model list of essential medicines for the treatment of nicotine dependence as an aid to stopping smoking and tobacco use. https://cdn.who.int/media/docs/default-source/2025-eml-expert-committee/addition-of-new-medicines/a.8_cytisine.pdf?sfvrsn=5df4226_6

40 World Bank Consortium: The Big Questions in Forced Displacement and Health. Jordan Country Report June 2022. Available from https://documents1.worldbank.org/curated/en/099001006212236284/pdf/P166909056ee1e0c08dfc098ebd3939849.pdf

41 Ministry of Health, Jordan. Smoking Cessation Clinics. Available from https://www.moh.gov.jo/AR/Pages/smoking.

42 Ministry of Health, Jordan Adult Tobacco Survey 2025. Factsheet. Available upon request.

43 Jordan National Stepwise Survey (STEPs) for Noncommunicable Diseases Risk Factors, Jordan 2019. https://www.moh.gov.jo/Echobusv3.0/SystemAssets/5f91caaf-c257-4806-837c-b1028147b692.pdf

44 Ministry of Health, Jordan Adult Tobacco Survey 2025. Factsheet. Available upon request.
