# Supplementary material for: Improving implementation of tobacco dependence treatment practice in low and middle-income countries settings: a perspective from Jordan
Source: Front Health Serv. 2025 Dec 17;5:1696442. doi: 10.3389/frhs.2025.1696442 (PMC12753926; doi:10.3389/frhs.2025.1696442)
Supplement: Supplementary file 1 [file Datasheet1.pdf]

Supplemental Table 1: Expert Recommendations for Implementing Change (ERIC) implementation strategies employed in tobacco dependence treatment efforts (TDT) in Jordan.

ERIC Domain	ERIC Implementation Strategy	Implementation in Jordan
**Train and educate stakeholders**	Conduct ongoing training	KHCC conducts annual certifiable training through its accredited training program. The program has been used by the majority of TDT practitioners in the country.
	Develop educational materials	Tools within KHCC training program are developed, and TDT guidelines are available.
	Make training dynamic	KHCC's training program is interactive.
	Distribute educational materials	Educational materials targeting cancer patients as well as general community developed and distributed across social media and through brochures. More novel platforms are currently being considered.
	Conduct educational meetings	Educational meetings frequently conducted within facility and across community, by staff involved in TDT (implementers and innovation deliverers); shorter training courses cover brief advice and the importance of TDT.
	Create a learning collaborative	Current local collaboratives are not available but ATTUD[1] offers an accessible platform KHCC is enrolled in.
**Provide interactive assistance**	Facilitation	In KHCC, a system involving medical staff (including Nursing and Pharmacy), the Patient Journey Office and the Quality Office ensures that interactive problem solving takes place when needed to improve or streamline patient access to TDT.
	Provide local technical assistance	The CCO[2] at KHCC supports the TDT clinic to facilitate implementation issues.

ERIC Domain	ERIC Implementation Strategy	Implementation in Jordan
**Use evaluative and iterative strategies**	Assess for readiness and identify barriers and facilitators	To varying degrees, assessments have been conducted but could be improved to become more regular.
	Audit and provide feedback	Conducted annually through the Quality Office at KHCC, but audits in other entities are less structured.
	Develop and implement tools for quality monitoring	Data collected across clinics but variability in scope and extent of use of data exists.
	Develop and organize quality monitoring systems	Data collected across clinics but variability in scope and extent of use of data exists. At KHCC, this data is currently being transformed to a more efficient tracking of TDT services.
	Conduct local need assessment	To varying degrees, needs assessments have been conducted, but could be improved to become more participatory and regular.
	Obtain and use patients/consumers and family feedback	Testimonials have been used in the TDT community across entities.
**Support clinicians**	Facilitate relay of clinical data to providers	The TDT clinic at KHCC collects data which it then utilizes to generate aggregate measures of TDT service performance as well produce research. More recently, data will be reflected in a dashboard for more timely tracking of key measures of performance.
**Utilize financial strategies**	Fund and contract for the clinical innovation	At the MoH, a grant from the WHO facilitated expansion of its TDT clinics.
	Place innovation on fee for service lists/formularies	Nicotine replacement therapies and varenicline placed on Jordan's Rational Drug List.
	Alter incentive/allowance structures	Employees at KHCC are offered free TDT
	Make billing easier	In situations where cash-payers are accessing the service; the billing process is streamlined.
	Develop disincentives	Smoking status is included in annual evaluations of employees at KHCC.

ERIC Domain	ERIC Implementation Strategy	Implementation in Jordan
**Develop stakeholder interrelationships**	Identify and prepare champions	Several champions exist within each entity to promote the value of TDT.
	Conduct local consensus discussions	Occasional meetings may take place within the TDT provider community, but more needs to be done to ensure these are more regular.
	Capture and share local knowledge	Local TDT experiences are shared through the training program at KHCC, which also encourages sharing of information and experience among local participants. However, this can be improved by being more regular a mechanism.
**Engage consumers**	Involve patients/consumers and family members	Testimonials from patients used in some platforms (events, video).
	Increase demand	The MoH, KHCC and others in the TDT community (college TDT clinic staff and Municipal TDT clinic staff) also conduct outreach to promote TDT.
	Use mass media	Frequently done across all entities to promote TDT service.
**Adapt and tailor to context**	Tailor strategies	Strategies tailored to cultural norms and language, and to patient needs.
**Change infrastructure**	Mandate change	On a national level, the creation of a tobacco control strategy which explicitly includes TDT-related objectives. At KHCC, more recent policies include a role for specific staff (e.g. clinic nurse coordinators) in screening for risk factors such as tobacco use, and intervening (brief advice).
	Change record systems	KHCC's EMR[3] has been modified to include TDT-related measures.
	Change physical structure and equipment	Purchase of CO monitors; clinic rooms available in Municipal clinic sites.
	Create or change credentialing and/or licensure standards	KHCC leveraged special import licensure to purchase TDT medications that were not yet nationally registered in Jordan.

1 Association for the Treatment of Tobacco Use and Dependence (ATTUD)

2 Cancer Control Office

3 Electronic Medical Record
